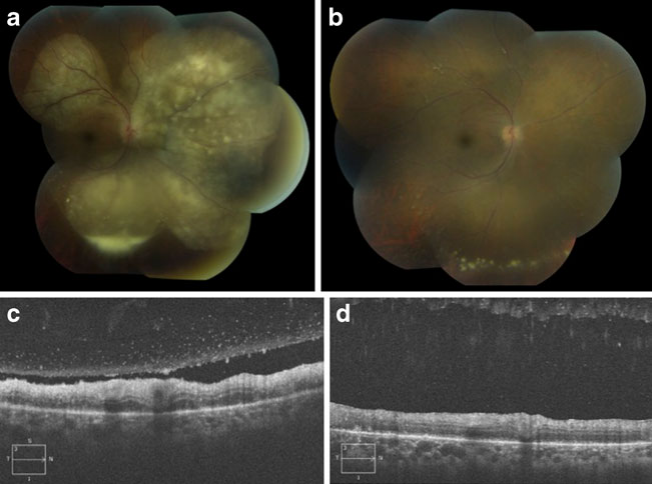# Diffuse infiltrative syphilitic retinitis in an HIV-positive patient

**DOI:** 10.1007/s12348-011-0026-x

**Published:** 2011-06-26

**Authors:** Hassan Toufiqur Rahman, Steven Yeh

**Affiliations:** Emory Eye Center, Emory University School of Medicine, 1365B Clifton Rd NE, Atlanta, GA 30322 USA

## Legend

A 40-year-old HIV-positive male (CD4 count 228 cells/ul) presented with decreased vision to 20/60 OD and hand motions OS. Funduscopic examination OD revealed 2+ vitritis with widespread intraretinal whitening, fluffy preretinal vitreous opacities, and a subhyaloidal hypopyon (a). A 4+ severe vitritis precluded retinal examination OS. PCR testing of aqueous fluid OD was negative for HSV, VZV, CMV, and toxoplasmosis DNA. Serum RPR and FTA-ABS were positive, and intravenous penicillin-G (four million units every 4 h) was initiated. Three weeks later, dramatic resolution of the vitreous opacities and retinitis was observed (b). Spectral domain OCT showed decreased macular thickening, disappearance of the vitreous opacities, and restoration of the inner retinal architecture from the initial visit (c) to the final follow-up visit (d).